# Clinical evaluation of toll-like receptor-5 agonist for radiation-induced oral mucositis in beagle dogs

**DOI:** 10.3389/fvets.2022.839467

**Published:** 2022-08-12

**Authors:** Jaeeun Ko, Jaehwan Kim, Yang-Kyu Choi, Sang-Soep Nahm, Jayon Kim, Sun-Min Seo, Jin-Seok Seo, Woojong Lee, Weon Kuu Chung, Kidong Eom

**Affiliations:** ^1^Department of Veterinary Medical Imaging, College of Veterinary Medicine, Konkuk University, Seoul, South Korea; ^2^Department of Laboratory Animal Medicine, College of Veterinary Medicine, Konkuk University, Seoul, South Korea; ^3^Department of Anatomy, College of Veterinary Medicine, Konkuk University, Seoul, South Korea; ^4^Connext Co. Ltd., Daegu, South Korea; ^5^Department of Radiation Oncology, Kyung Hee University Hospital at Gangdong, Seoul, South Korea

**Keywords:** radiotherapy, radiation countermeasure, toll-like receptor-5, flagellin, dog

## Abstract

This study aimed to evaluate the clinical safety and validate the radiomitigative effect of KMRC011, against radiation-induced oral mucositis in beagle dogs. Clinical safety was evaluated by assessing tolerability, complete blood tests, and plasma biochemistry after drug administration. The radiomitigative effect of KMRC011 was evaluated macropathologically and histopathologically after inducing oral mucositis iatrogenically using 20 Gy irradiation. The plasma concentration of interleukin-6 was measured *via* enzyme-linked immunosorbent assay, as a biomarker of KMRC011 bioreactivity. Decreased tolerability, increased neutrophil count, hepatic enzyme concentration, C-reactive protein concentration, and interleukin-6 concentration after the administration was observed and ceased within 24 h without additional treatment. Although all animals included in the present study developed severe mucositis in the late course of the study, animals administered KMRC011 showed less erythema, ulcer, inflammatory infiltration. These results suggest that KMRC011 may be used as an adjuvant for radiotherapy without severe adverse effects, especially during short-term radiotherapy, such as hypofractionated radiotherapy or stereotactic radiotherapy.

## Introduction

Oral mucositis, one of the gastrointestinal manifestations of radiation exposure, was first described in the late 1980s as cytotoxicity in the oral mucosa of cancer patients receiving anti-cancer therapy (1, 2). Oral mucositis occurring due to radiotherapy (RTx) is explicitly referred to as radiation-induced oral mucositis (RIOM). The onset of RIOM occurs during the early course of RTx when cumulative exposure reaches 15 Gy and drastically worsens when total accumulation exceeds 60 Gy (3). Although RIOM is relatively well managed through appropriate treatment and is resolved within 2–4 weeks after cessation of RTx, RIOM remains the most critical manifestation in cancer patients (4–6). Regardless of the favorable prognosis of RIOM, the quality of life of patients with RIOM is severely compromised owing to pain, which is the most significant symptom (7). Pain from the injured mucosa contributes to lower food intake and subsequent malnutrition, extended hospitalization, and even requires reduction or cessation of RTx, which in turn compromises tumor control (4, 8–10). Thus, although RIOM is a milder, relatively less life-threatening complication of RTx, there is an urgent need for radiation countermeasures in cancer patients to improve their quality of life and extend the therapeutic window of RTx.

Multiple agents for the alleviation of RTx-associated complications are at the clinical trial stage, but only a few agents, such as amifostine, are commercially available. Entolimod, a toll-like receptor (TLR)-5 agonist, is also being assessed as a radiation countermeasure in clinical trials. The most frequently observed adverse event of Entolimod was flu-like syndrome, which is characterized by chill, fever, and head and body ache similar to real flu. However, this phenomenon was consistent with the mechanism of action of the drug and was not considered as a clinically relevant complication (11). The mechanism of TLR-5 agonists as a radiation countermeasure is mediated *via* the activation of multiple transcription factors, including NF-κB, which stimulates a cascade of cell signaling pathways to promote anti-acute radiation syndrome effects before or right after radiation damage. Major cytoprotective factors activated by TLR-5 agonists are interleukin (IL)-6, granulocyte colony-stimulating factor, superoxide dismutase 2, and anti-apoptotic proteins (12).

The possible drawback of Entolimod to use in clinical practices comes from its innate molecular structure, the histidine tag. These 33 amino acid residues are “labels” of flagellin proteins to identify and purify Entolimod during manufacturing (13). Although histidine itself is known not to have immunogenicity, there is concern that binding with other molecules can provide unexpected immunogenicity and decrease the binding ability of the drug (14). To overcome this disadvantage, the KMRC011, the flagellin without a histidine tag, was developed recently (Korea Institute of Industrial Technology, Yeongcheon, Korea). Its ability as a TLR-5 agonist and radiation countermeasure was also reported using mice and cynomolgus monkeys (15–17).

Over the past decades, RTx has become more common and has rapidly evolved as a standard of treatment for oncologic patients in veterinary medicine (18). Considering the usage of RTx in modern veterinary medicine, RIOM has not been as extensively studied in animals as in humans, with RIOM reports usually provided as supplemental information in RTx case reports (19). Nevertheless, RIOM is common among veterinary patients undergoing RTx and thus requires attention as well as improved treatment.

To date, there are no available data on the radioprotective ability of KMRC011 in dogs. Despite its structure being near-identical to that of Entolimod, to assess the applicability of KMRC011 in dogs, its efficacy as a TLR-5 agonist should be evaluated. Thus, in the present study, we sought to analyze the applicability of KMRC011 as an adjuvant for RTx in dogs, hypothesizing that KMRC011 will mitigate radiation damage to the oral cavity.

## Materials and methods

### Animals and ethical approval

A total of six dogs were purchased for this study. Dogs were kept at the College of Veterinary Medicine, Konkuk University, under normal room temperature and humidity conditions. Regular feed was provided as per the manufacturer's recommendation (Nestlé Purina PetCare Company, Ringwood, NJ, USA), with free access to water. All animals were acclimated for at least 7 days before starting the experiments. The condition of animals during the acclimation period was evaluated by physical examination, thoracic and abdominal radiographs, abdominal ultrasonography, echocardiography, complete blood count (CBC), and plasma biochemistry to confirm animal normality. Recorded data from the acclimation period were also used as a baseline. Dogs were randomly allocated into two groups: control (*n* = 3) and treatment group (*n* = 3). This study was approved by the Institutional Animal Care and Use Committee of Konkuk University (KU21029).

### Irradiation of animals

Animals were anesthetized with a dose of 0.6 ml/kg propofol (Anepol; HaNa Pharm, Seoul, Korea) and 2% isoflurane. Before irradiation, computed tomographic (CT) simulations were performed (Brilliance Big Bore CT scanner; Philips Medical Systems, Cleveland, OH, USA). Dogs were positioned in ventral recumbency with a vacuum-immobilization cushion. Only pre-contrast CT scans were acquired. Field setting and dose prescription were performed with a commercial treatment planning system (Eclipse; Varian Medical Systems, Palo Alto, CA, USA). The planning target volume was defined as a 2 × 2 cm rectangular field from the 1st canine to the 4th premolar teeth, centered in the oral cavity. Irradiation was performed by a linear accelerator (Varian iXTM; Varian Medical Systems, Palo Alto, CA, USA). Using the conventional four-field box technique, 20 Gy of radiation was prescribed. Position verification was performed with an on-board cone-beam CT. If necessary, the dogs' position was adjusted and cone-beam CT was repeated for position verification. Radiation was delivered by the setting of 6 MeV photons with 600 monitoring units per minute setting.

### KMRC011 preparation and administration

KMRC011 was provided as a freeze-drying powder with a total dose of 150 μg/vial (Korea Institute of Industrial Technology, Yeongcheon, Korea). KMRC011 was prepared as per the manufacturer's manual. 3 mL of distilled water was injected into the vial to prepare a 50-μg/ml solution. All drugs were dissolved just before use, and the remaining volumes were disposed. KMRC011 was administered through the biceps femoris.

20 μg/kg of KMRC011 was administered three times to the treatment group: right after irradiation, 24 h after irradiation, and 48 h after irradiation. Since provided dry powder was constituted with flagellin protein and buffer, normal saline was used for the control group.

### Evaluation of the radiomitigative effect of KMRC011

The radiomitigative effect of KMRC011 was assessed *via* macropathological and histopathological evaluation. For macropathological assessment, bilateral maxilla, mandible, and tongue were photographed once a day from day 0 to 14, and once a week from day 14 to 56. The extent of erythema and ulcer or area covered with pseudomembrane was scored semi-quantitatively as shown in Table 1.

**Table 1:** Criteria for macropathologic scoring of RIOM.

**Erythema/ulcer coverage percent**	**Score**
No changes over baseline	0
>25% of mucosal surface	1
25–50% of mucosal surface	2
>50% of mucosal surface	3

For histopathological assessment, buccal oral mucosal tissue was obtained 9 and 56 days after irradiation using a 6-mm punch biopsy (Disposable Biopsy Punch; Kai Medical, Solingen, Germany). Biopsy sites were selected randomly from the inner surface of the right upper lip between the level of the first canine to the fourth premolar. Obtained tissue samples were fixated in a 10% neutral buffered formalin solution for 24 h. After formalin fixation, the samples were processed and embedded in paraffin blocks as per routine procedures and stained with hematoxylin (Harris Hematoxylin; YD Diagnostics, Yong-In, Korea) and eosin (Eosin Y Alcoholic; BBC biochemical, Washington, DC, USA). The maximal and minimal epithelial height as well as the ratio of inflammatory infiltration area in lamina propria to the total area in the lamina propria (Inflamm:LP) were determined under an optical microscope using Image J software (20).

### Enzyme-linked immunosorbent assay for IL-6

The concentration of IL-6 in peripheral blood was analyzed in serum samples obtained at 0, 1, 2, 4, 8, and 24 h after the administration. Blood samples (3 ml) were collected from the external jugular vein, immediately transferred to a serum separation tube, and incubated for 30 min at room temperature. After incubation, serum was separated *via* centrifugation. Separated serum was aliquoted into 250 μl and stored at −70°C until use. A commercially available ELISA kit was used for analysis (CA6000, R&D Systems, Minneapolis, MN, USA). The assay was performed as per the manufacturer's instructions. The samples were diluted when the optical density was too high, and the assay results were multiplied by the dilution factor. IL-6 concentrations under the limit of detection were recorded as 0 to facilitate analysis. The peak concentrations of IL-6 after each administration in the treatment group and IL-6 concentration in the control group at the corresponding timepoint were used for analysis.

### Tolerability scoring

The tolerability of animals at each timepoint was scored on a 0–3 scale. Scoring was based on the physiological status of animals, that is, lethargy and algor. Lethargy was divided into two categories: response to exogenous stimuli (e.g., sound, smell) and anergy to exogenous stimuli. Algor was divided into two categories: with hyperthermia and without hyperthermia. Body temperature (BT) was recorded at 0, 1, 2, 4, 8, and 24 h after administration. BT was measured rectally three times using a thermometer, and the mean BT was used for analysis. Tolerability was recorded comprehensively in a blinded manner as follows: 0, no changes over baseline; 1, tolerable without supportive care; 2, tolerable but supportive care required; and 3, not tolerable, need to stop the experiment and intensive care required (Table 2).

**Table 2:** Criteria for tolerability scoring.

**Criteria**	**Score**
No changes over baseline	0
Tolerable without supportive care	1
Tolerable but animal supportive care required	2
Not tolerable, need to stop the experiment and intensive care required	3

### CBC and plasma biochemistry examination

CBC and plasma biochemistry were performed 2 and 6 h after the administration during clinical safety evaluation. Peripheral blood (1.5 ml) was collected from the external jugular vein and immediately transferred to an ethylenediaminetetraacetic acid-treated sample tube for CBC analysis (0.5 ml) and a heparinized sample tube for plasma biochemistry (1 ml). Parameters related to cytokine release syndrome, including hematocrit (HCT), white blood cell count (WBC), neutrophil count (NEU), C-reactive protein (CRP), alanine aminotransferase (ALT), and alkaline phosphatase (ALP), were analyzed with a hematology analyzer (Procyte One Hematology Analyzer, IDEXX Laboratories, Westbrook, ME, USA) and a chemistry analyzer (Catalyst One Chemistry Analyzer, IDEXX Laboratories). All samples were analyzed right after collection.

### Statistical analysis

Data were collected and analyzed using SPSS Statistics, version 26.0 (IBM SPSS Inc., Chicago, IL, USA). All data are expressed as the mean ± standard deviation. Tolerability, body temperature, CBC parameters and plasma biochemistry parameters were analyzed using the Friedman test to compare the collected parameters between timepoints after each administration. *Post-hoc* analyses were performed *via* pairwise comparison with a Bonferroni correction. In tolerability assessment scores, inter-investigator reliability was analyzed by the interclass correlation coefficient (ICC), which was calculated before further analyses. ICC were graded as follows: ICC > 0.8, excellent; 0.8 > ICC > 0.6, very good; 0.6 > ICC > 0.4, good; 0.4 > ICC > 0.2, questionable; 0.2 > ICC > 0, unacceptable. The Mann–Whitney U test was used to compare the peak concentration of IL-6 as well as macropathological and histopathological differences between the treated and control group. A *p*-value < 0.05 was considered to indicate significance.

## Results

### Elevation of IL-6 after the AKMRC011 administration

The treated group had significantly (*p* < 0.05) higher levels of IL-6 after each administration of KMRC011. IL-6 concentration in the treated group was 18745.19 ± 13052.85, 3643.25 ± 1046.16, and 1447.42 ± 77.13 after the first, second, and third administration, respectively, while IL-6 the corresponding values in the control group were 78.89 ± 30.79, 79.08 ± 49.48, and 47.06 ± 15.03 pg/mL (Table 3). Although the longitudinal statistical analysis between each timepoint was not performed, a gradual attenuation of IL-6 induced by the continuous administration of KMRC011 was also observed.

**Table 3:** Peak IL-6 concentration after the administration of KMRC011.

	**Peak IL-6 concentration (pg/ml)**	
**Day**	**Control group**	**Treated group**
1	78.89 ± 30.79	18745.19 ± 13052.85^*^
2	79.08 ± 49.48	3643.25 ± 1046.16^*^
3	47.06 ± 15.03	1447.42 ±77.13^*^

* p < 0.05.

### Mild to moderate radiomitigative effect of KMRC011

**Figure 1:**
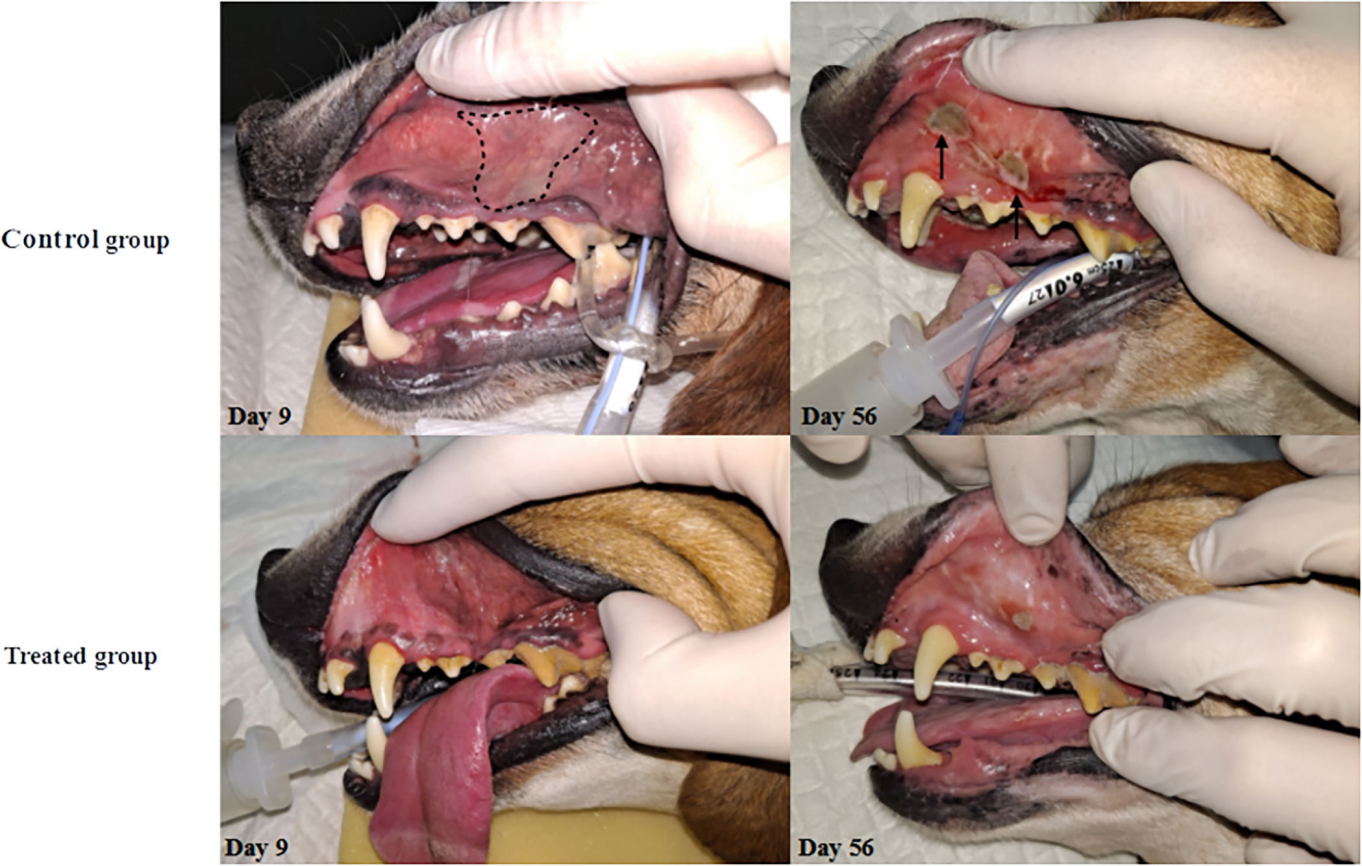
Changes in oral mucosa after irradiation. The onset of RIOM was observed on day 9 as a form of erythema on oral mucosa and the presence of pseudomembrane (whitish-discoloration). Notably, pseudomembrane was only observed in the control group on day 9 (dashed line). After the ulceration phase begins, the control group showed accelerated progress of the disease with bleeding and edematous changes of oral cavity mucosa (solid arrow) while the treated group only showed minor ulceration on day 56.

Macropathological assessment indicated a significantly lower (*p* < 0.05) extent of erythema (1.3 ± 0.1) and ulcers (0.5 ± 0.4) in the treatment group than in the control group (erythema, 2.5 ± 0.1; ulcer 1.5 ± 0.8) at 9 days after irradiation. Fifty-six days after irradiation, only erythematous lesions were significantly (*p* < 0.05) lower in the treatment group (0.7 ± 0.4) than in control group (1.7± 0.5) (Figure 1, Table 4).

**Table 4:** Macropathological score of RIOM.

	**Days after**	**Control**	**Treated**
	**irradiation**	**group**	**group**
Erythema	9 days	2.5 ± 0.1	1.3 ± 0.1^*^
	56 days	1.7± 0.5	0.7 ± 0.4^*^
Ulcer	9 days	1.5 ± 0.8	0.5 ± 0.4^*^
	56 days	0.7 ± 0.1	0.5 ± 0.3

* p < 0.05.

**Figure 2:**
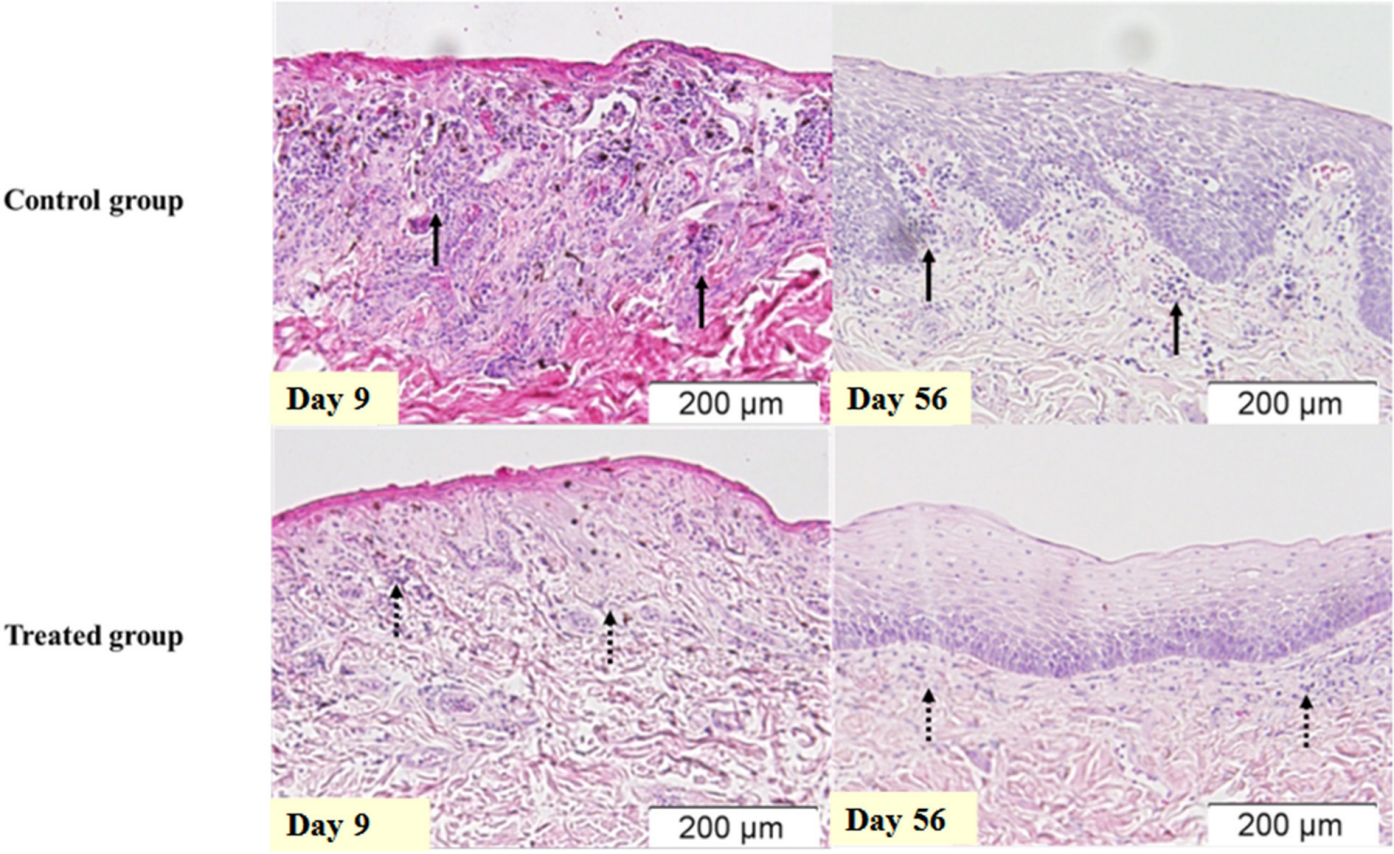
Inflammatory infiltration of the oral mucosa after irradiation. Oral mucosa of same dogs in Figure 1, stained with hematoxylin and eosin. Infiltration of inflammatory cells is more prominent in the control group (solid arrow) when compared to the treated group (dashed arrow).

The histopathological assessment indicated that only Inflamm:LP has significant differences (*p* < 0.05). Inflamm:LP at 9 days after irradiation were 6.2 ± 1.7 and 10.1 ± 2.0 in treated and control group, respectively. No significant difference was observed in maximum and minimum epithelial thickness. This result was consistent in tissue specimens obtained 56 days after irradiation, with 2.2 ± 0.3 of Inflamm:LP in treated group and 4.6 ± 1.2 of Inflamm:LP in control group (Figure 2, Table 5).

**Table 5:** Histopathological score of RIOM.

	**Days after**	**Control**	**Treated**
	**irradiation**	**group**	**group**
Max. epi. thickness (μm)	9	27.3 ± 47.3	0
	56	298.9 ± 86.2	229.3 ± 77.4
Min. epi. thickness (μm)	9	0	0
	56	151.9 ± 76.5	99.3 ± 16.8
Inflamm:LP	9	10.1 ± 2.0	6.2 ± 1.7^*^
	56	4.6 ± 1.2	2.2 ± 0.2^*^

Max. Epi. thickness, maximal epithelial thickness; Min. Epi. thickness, minimal epithelial thickness; Inflamm:LP, inflammatory infiltration area to lamina propria area ratio.

* p < 0.05.

### Acute inflammatory response after KMCR011 administration (flu-like syndrome)

**Figure 3:**
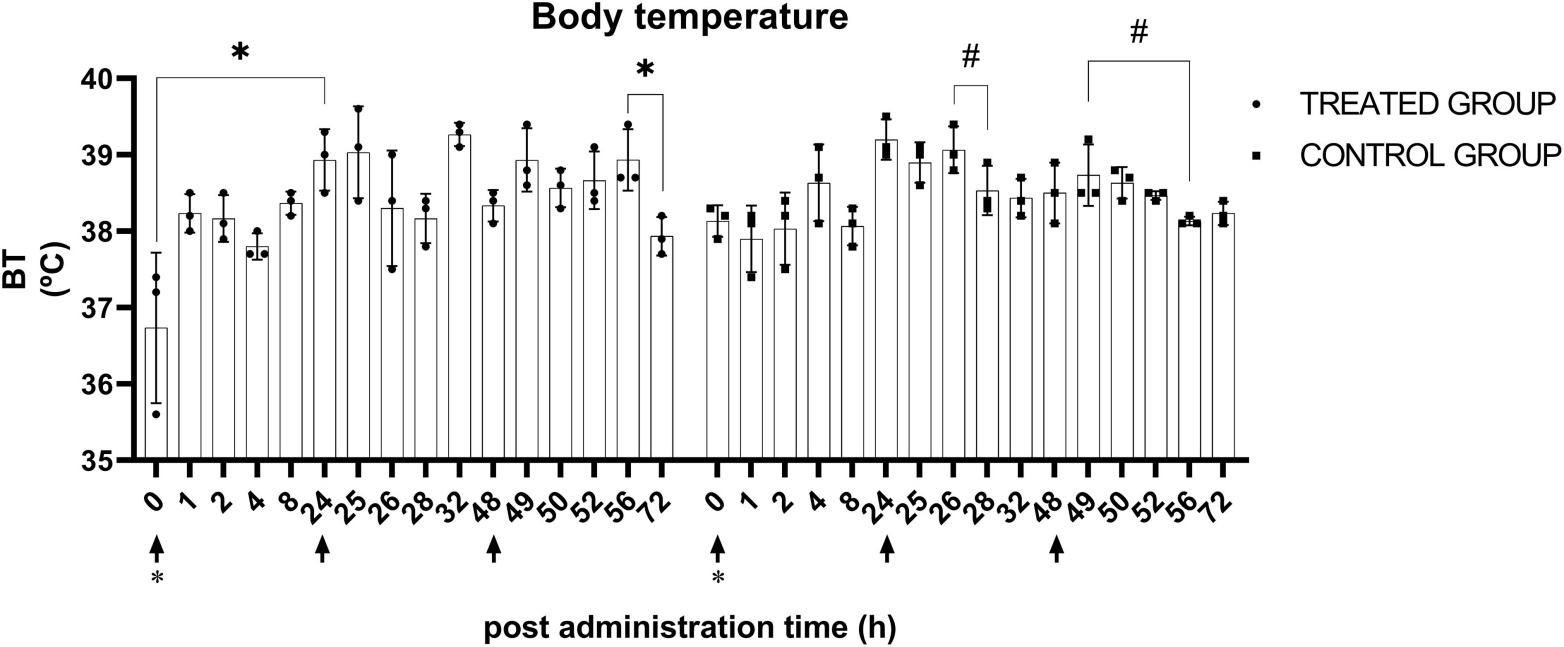
Body temperature after KMRC011 or normal saline administration. KMRC011 was administered (solid arrows) at 0, 24, and 48 h after irradiation (asterisk). Body temperature showed significant changes in both control and treated groups after the administration. However, there was no consistency and none of the animals showed actual hyperthermia. *p < 0.05; ^#^*p* < 0.05, largest difference without statistical significance in the *post-hoc* analysis.

After the first and third administration, there were significant BT changes (*p* < 0.05) in the treatment group. The BT at 0 h after the first administration (36.7 ± 0.9°C) and the BT at 24 h after the first administration (38.9 ± 0.4°C) were significantly (*p* < 0.05) different. After the third administration, no significance was determined *via post-hoc* analysis, while at 8 h (38.9 ± 0.4°C) and 24 h after the administration (37.9 ± 0.3°C), the most prominent differences were observed between each timepoint. There were significant (*p* < 0.05) BT changes in the control group after the second and third administration of normal saline. BT at 2 h after the second administration (39.1 ± 0.3°C) and 24 h after the administration (38.5 ± 0.4°C) exhibited the largest difference, but no significance was determined in *post-hoc* analysis. After the third administration, no significance was identified *via post-hoc* analysis, but the BT at 0 h (38.7 ± 0.2°C) and 8 h (38.1 ± 0.1°C) after administration exhibited the largest differences between each timepoint. Data plotted by timepoint are shown in Figure 3.

**Figure 4:**
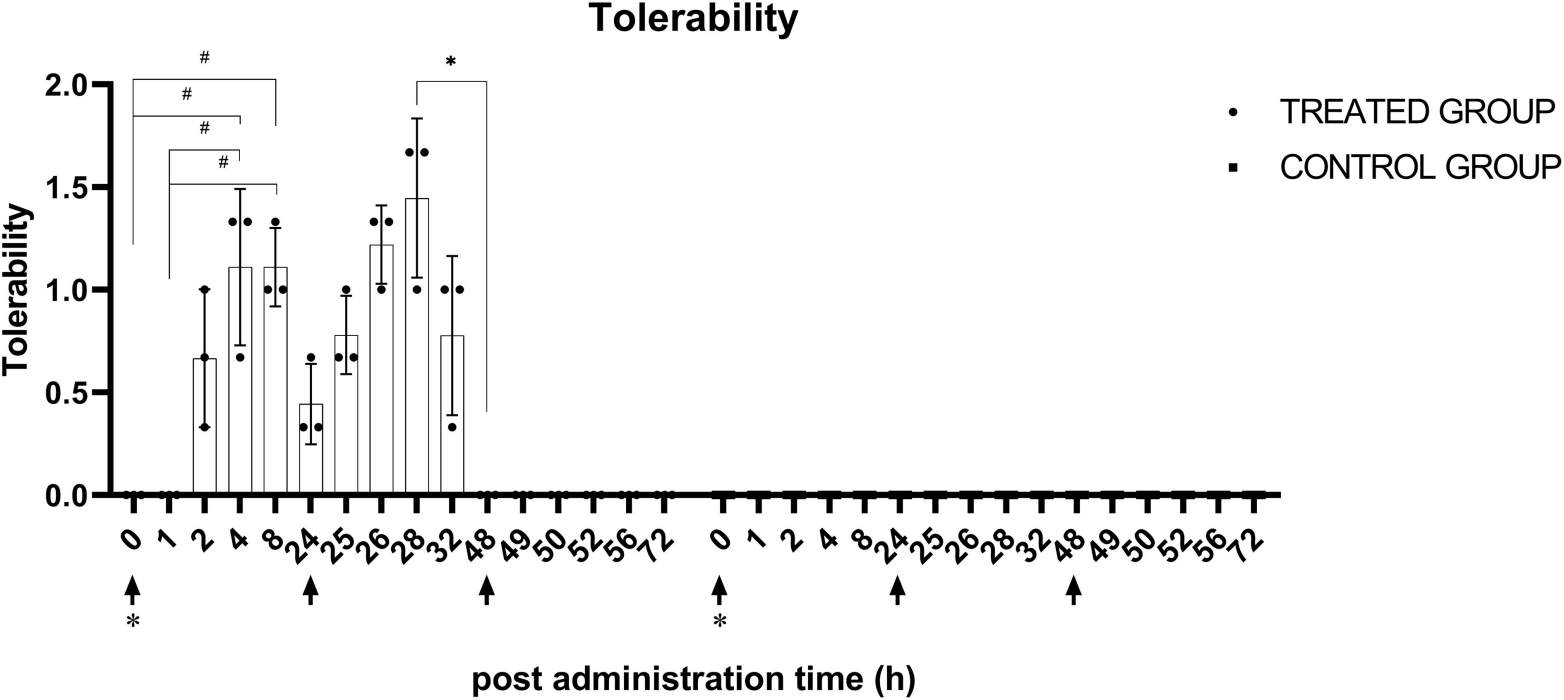
Tolerability score after KMRC011 or normal saline administration. KMRC011 was administered (solid arrows) at 0, 24, and 48 h after irradiation (asterisk). Tolerability score in the treated group showed significant elevation within 1–2 h after the first and second administration and decreased within 4–8 h after the administration. After the third administration, there were no changes in tolerability score. The Control group showed no observable changes after the administration. *p < 0.05; ^#^*p* < 0.05, largest difference without statistical significance in the *post-hoc* analysis.

The inter-investigator reliability in tolerability evaluation appeared to be excellent, with an ICC of 0.82 between investigators. There were significant (*p* < 0.05) changes in tolerability score in the treatment group after the first and second administration of KMRC011. There were no statistically significant differences between each timepoint after the third administration of KMRC011. The largest differences were identified between 0 h (0) and 4 h after the administration (1.1 ± 0.4), 0 h (0) and 8 h after the administration (1.1 ± 0.2), and 1 h (0) and 4 h after the administration (1.1 ± 0.4), as well as 1 h (0) and 8 h after the administration (1.1 ± 0.2). After the second administration of KMRC011, the tolerability score at 4 h after administration (1.4 ± 0.4) was significantly higher than that at 24 h after the administration (0). There were no significant differences between each timepoint after the third administration of KMRC011. In the control group, no observed changes occurred after each administration of normal saline. The data plotted by timepoint are shown in Figure 4.

**Figure 5:**
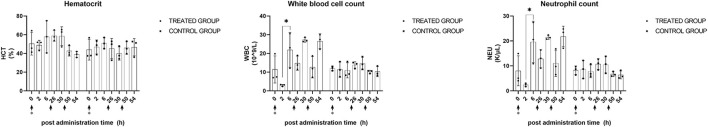
CBC profiles after KMRC011 or normal saline administration. KMRC011 was administered (solid arrows) at 0, 24, and 48 h after irradiation (asterisk). WBC and NEU showed significant changes after the first administration while no observed statistical significance after the second and third administration. HCT, hematocrit; WBC, white blood cell count; NEU, neutrophil count; *p < 0.05.

In CBC, only the treated dogs exhibited statistically significant changes after KMRC011 administration. The WBC was significantly elevated (*p* < 0.05) 6 h after the first administration (21.9 ± 9.3 × 10^9^/L) compared to 2 h after the administration (3.0 ± 0.6 × 10^9^/L). NEU at 6 h after the administration (19.6 ± 8.3 K/μl) was significantly (*p* < 0.05) higher than that at 2 h after the administration (2.4 ± 0.7 K/μl). The data plotted by timepoint are shown in Figure 5.

**Figure 6:**
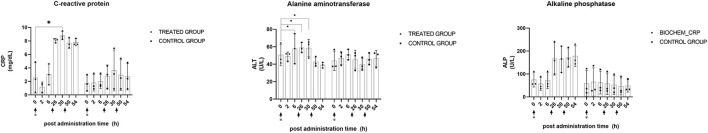
Plasma biochemistry profiles after KMRC011 or normal saline administration. KMRC011 was administered (solid arrows) at 0, 24, and 48 h after irradiation (asterisk). Only the treated group showed significant changes in plasma biochemistry profiles. ALT significantly changed after the first and second administration and CRP significantly changed after the second administration. ALP did not show significant changes after the administration. CRP, C-reactive protein; ALT, alanine aminotransferase; ALP, alkaline phosphatase; CRP, C-reactive protein; *p < 0.05.

CRP and ALT exhibited statistically significant (*p* < 0.05) differences in the treatment group. ALT levels showed a significant (*p* < 0.05) increase after the first and second administration of KMRC011. Six hours after the first administration, ALT was significantly (*p* < 0.05) increased (110.3 ± 32.9 U/L) compared to that at baseline (42.3 ± 5.5 U/L). Two hours after the second administration, ALT still showed a significant (*p* < 0.05) increase (75.0 ± 16.5 U/L) compared to that at baseline. The CRP level measured 6 h after the second KMRC011 administration (8.8 ± 0.6 mg/dl) was significantly (*p* < 0.05) increased compared to that at baseline (2.5 ± 2.3 mg/dl). A statistically significant increase in ALT was also observed in the control group. ALT levels 6 h after the second administration (51.7 ± 17.0 U/L) were significantly (*p* < 0.05) higher than those at baseline (24.7 ± 13.4 U/L). The data plotted by timepoint are shown in Figure 6.

## Discussion

In the present study, KMRC011 showed early radioprotection against 20 Gy irradiation in the oral cavity without causing clinically significant adverse effects. Treated dogs exhibited less erythema, ulcers, and inflammatory infiltration in the oral cavity at 9 days after irradiation as well as less erythema and inflammatory infiltration at 56 days after irradiation compared to the control group.

Elevation of the WBC, neutrophilia, CRP, and hepatic enzymes, as well as the flu-like syndrome, were observed in the present study. Previous reports of this complication in humans described more prominent adverse effects than those observed in dogs in the present study (21). Structural differences in TLR-5 between the two species have been reported, although both receptors serve the same role within immunity (22). Although between-species differences may have functional significance in the response to KMRC011 administration, we could not assess this based on the available data.

KMRC011 successfully induced elevation of IL-6 levels in serum within at least 4 h after administration. Elevation of IL-6 by irradiation was previously reported (23). However, the effect of radiation on IL-6 levels can be ignored, since the concentration of IL-6 in the treatment group was significantly (*p* < 0.05) higher than that in the control group. In the present study, the IL-6 concentration was different from previously reported values in Entolimod-treated dogs (20). A 5-μg/kg dose of KMRC011 induced an IL-6 concentration equivalent to that achieved under a 3- to 10-μg/kg dose of Entolimod. However, it cannot be inferred that KMRC011 has better efficacy than Entolimod due to differences in study design, the small number of samples, and the large standard deviation, indicating that individual differences primarily affect efficacy. Therefore, further investigation is needed to compare the effects of KMRC011 and Entolimod.

The attenuation of the peak concentration of IL-6 after the administration of KMRC011 was newly observed in the present study. The attenuation of KMRC011 bioreactivity was not considered adaptive immunization since this phenomenon appeared in a relatively short period (~48 h). The rapid development of tolerance to flagellin was previously reported in polarized intestinal epithelial cells (24). Prolonged flagellin exposure to polarized epithelial cells inhibits the activation of NF-κB *via* internalization of TLR-5 and occurs within 1 to 2 h of flagellin exposure. Over 24 h were necessary to restore TLR-5 expression. Although this tolerance phenomenon was observed under *in vitro* conditions, the rapidly attenuated response to KMRC011 in the present study supports the report that such tolerance may also occur *in vivo*.

Although the peak concentration level of IL-6 gradually decreased under prolonged KMRC011 administration, IL-6 was sufficiently elevated after three sequential administrations of KMRC011 to achieve statistical significance. There is controversy regarding whether radioprotective efficacy is decreased or not by previous exposure to TLR-5 agonists (11). Multiple investigations from the Entolimod manufacturer demonstrated that its administration rapidly induced the production of anti-flagellin antibodies in 2–3 weeks. As Salmonella infection can occur naturally, the presence of antibodies was also reported in subjects who were not exposed to Entolimod. While these reports describe the need for multiple administrations of Entolimod to protect against multiple radiation exposures, another report highlights the capacity of single Entolimod administration as sufficient against multiple radiation exposures (13). This discrepancy may be due to differences between the concentration levels of IL-6 in systemic circulation and local tissue. Following the repeated administration of TLR-5 agonists, IL-6 concentration in local tissue may facilitate radioprotective cell signaling, even though there is attenuation of induction of IL-6 by KMRA011. In the above-described study, Entolimod exhibited its radioprotective ability in mice which was administered the drug over five sequential days. The present findings partially support the above-mentioned study, as KMRC011 bioreactivity was retained over sequential administration for 3 days.

The current study has a few limitations. The sample number was small, only a single radiation dose was used, and the molecular mechanism of KMRC011 was not explored in the oral mucosal epithelia. A large number of samples with multiple radiation doses were not considered due to ethical reasons. Nevertheless, we observed a partial radiomitigative effect of KMRC011 against 20 Gy irradiation. It is suggested that KMRC011 can mitigate radiation injury more effectively at a smaller radiation dose, such as the conventionally used radiation dose in RTx. With regard to the molecular mechanism underlying the effects of KMRC011, we tried to determine NF-κB changes in tissue specimens collected 9 days after irradiation but failed to do so, as most mucosal epithelial cells were lost. Signaling related to the activation of TLR-5 is known to occur before the visible onset of RIOM, which means that NF-κB may not be detectable after RIOM onset. To evaluate this, a biopsy would have to be performed earlier, which was not considered in the present study, since a biopsy itself can accelerate RIOM development by inducing inflammation. The current study aimed to determine the radiomitigative effect of KMRC011 in dogs in the clinical setting. We achieved this end goal *via* CRI, macropathological, and histopathological assessment, utilizing tissue biopsy as per a previously reported method for the investigation of iatrogenically induced RIOM (25). The distribution of IL-6 within the oral cavity was not analyzed. There is controversy regarding the relationship between systemic and local cytokine levels (26, 27). However, in a previous study, KMRC011 successfully protected mouse intestinal epithelia from radiation injury under elevated systemic IL-6 levels after KRMC011 administration (15). Therefore, elevated serum IL-6 was believed to represent the activation of TLR-5 at the local level, particularly in gastrointestinal epithelial cells.

In conclusion, using KMRC011 as an adjuvant can be helpful for cancer patients, especially when short-term RTx protocols are used.

## Data availability statement

The original contributions presented in the study are included in the article/supplementary material, further inquiries can be directed to the corresponding author/s.

## Ethics statement

The animal study was reviewed and approved by Institutional Animal Care and Use Committee of Konkuk University.

## Author contributions

JaeeK and JaehK: study design, contouring, statistical analysis, and irradiation. Y-KC and S-MS: pathological scoring and ELISA analysis. S-SN and J-SS: pathological scoring, specimen preparation, and staining. JayoK: contouring and animal care and examination. WL: study design (partly). WC and KE: study design, irradiation, and supervising. All authors contributed to the article and approved the submitted version.

## Conflict of interest

Author WL was employed by the company Connext Co. Ltd. The remaining authors declare that the research was conducted in the absence of any commercial or financial relationships that could be construed as a potential conflict of interest.

## Publisher's note

All claims expressed in this article are solely those of the authors and do not necessarily represent those of their affiliated organizations, or those of the publisher, the editors and the reviewers. Any product that may be evaluated in this article, or claim that may be made by its manufacturer, is not guaranteed or endorsed by the publisher.
